# A review of appropriate indicators for need-based financial resource allocation in health systems

**DOI:** 10.1186/s12913-021-06522-0

**Published:** 2021-07-09

**Authors:** Maryam Radinmanesh, Farbod Ebadifard Azar, Asgar aghaei Hashjin, Behzad Najafi, Reza Majdzadeh

**Affiliations:** 1 Department of Health Economics, School of Health Management and Information Sciences, Iran University of Medical Sciences, Tehran, Iran; 2 Department of Health Services Management, School of Health Management and Information Sciences, Iran University of Medical Sciences, Tehran, Iran; 3 Iranian Center of Excellence in Health Management, School of Management and Medical Informatics, Tabriz University of Medical Sciences, Tabriz, Iran; 4 Knowledge Utilization Research Center, Community-based Participatory Research Center and School of Public Health, Tehran University of Medical Sciences, Tehran, Iran

**Keywords:** Budget, Health resources, Needs assessment, Resource allocation

## Abstract

**Background:**

Optimal, need-based, and equitable allocation of financial resources is one of the most important concerns of health systems worldwide. Fulfilling this goal requires considering various criteria when allocating resources. The present study was conducted to identify the need indicators used to allocate health resources in different countries worldwide.

**Methods:**

A systematic review conducted on all published articles and reports on the need-based allocation of health financial resources in the English language from 1990 to 2020 in databases, including PubMed, Cochrane, and Scopus as well as those in Persian language databases, including magiran, SID, and Google and Google scholar search engines. After performing different stages of screening, appropriate studies were identified and their information were extracted independently by two people, which were then controlled by a third person. The extracted data were finally analyzed by content analysis method using MAXQDA 10 software.

**Result:**

This search yielded 823 studies, of which 29 were included for the final review. The findings indicated that many need-based resource allocation formulas attempt to deal with health care needs using some weighting methods for individuals. In this regard, the most commonly used indicators were found as follows: age, gender, socio-economic status or deprivation, ethnicity, standardized mortality ratio (SMR), the modified health indicators (disease consequences, self-assessed health, and disability), geographical area / place of residence (geographical) (rural versus urban), cross-boundary flows, cost of services, and donations.

**Conclusion:**

The indicators used in allocating the health systems’ financial resources in each country should be designed in order to be simple and transparent and in accordance with the moral norms of that society. Moreover, these should be a good representative of the health needs of people in different geographical areas of that country. In addition, their related data should be available to an acceptable extent.

## Introduction

Having access to health services with the aims of promoting, maintaining, and ensuring the individuals’ health is considered as one of the important pillars in the development of any society [1]. However, health systems are usually faced with the shortage of resources, so on the one hand, they are not able to provide all the services required for all society members [2] and on the other hand, they seek to increase justice in people’s access to health care and services [3]. Concerning limited financial resources, world health systems mostly face various challenges in terms of service quality, efficiency, effectiveness, and justice; therefore, equitable resource allocation is regarded as one of the major concerns of any health system in any part of the world and is also known as a key part of the decision-making process in this regard [4, 5]. As a moral issue, it plays critical roles in creating justice in health care services and in its health consequences [6]. Resource allocation is fair when health care resources are equally distributed among competing consumers (e.g. regions) based on their needs to health care [7]. Accordingly, need-based resource allocation is one of the methods that has been considered for equitable allocation of resources in recent decades in most of publicly financed health care systems [8–10]. Additionally, need-based resource allocation is a process in which financial resources are distributed among individuals or populations based on their needs to health care services [11, 12]. In fact, need-based resource allocation attempts to eliminate budget inequalities among the areas of a region or a country [13]. Using this approach, it can be ensured that government’s public resources are equally distributed to different regions in accordance with the goals of the health system [14]. Since it is difficult to measure health care needs in a society directly, so some indirect methods like indicators can be used to measure health needs [15]. Up to now, no standard gold indicator has revealed the need for health care services in communities. Consequently, this has caused several challenges in selecting the required indicators to compile need-based resource allocation formulas [16]. Therefore, the models and indicators used in this regard are significantly diverse [17].

According to the World Health Organization, in recent decades, health and treatment conditions have improved in most countries, and these improvements have been reflected in some indicators such as “life expectancy at birth, life expectancy with the balanced disability, and decreased mortality rate resulted from health measures”. However, these improvements have always been accompanied by some concerns, the most important of which are inequalities in having access to health care services and health consequences among people from different economic groups inside and outside countries [18, 19]. Therefore, concerning numerous challenges existing due to the shortage of resources to provide equitable health care services, the equitable allocation of available resources is of great importance. Correspondingly, this requires applying the need-based allocation method instead of the currently used resource allocation approaches. Since creating a suitable indigenous model requires studying the models of other countries for identifying their principles and the type of indicators used by them, the present study aimed to identify the indicators used for need-based resource allocation in health systems worldwide through conducting a comprehensive review on the related studies.

## Methods

### Study design and search strategy

This systematic review was done to answer the question of what models and indicators are mostly used for need-based resource allocation in the health systems worldwide. Therefore, to answer this question, a set of keywords was searched in English and Persian language databases, which are represented in Table 1. Thereafter, to find published academic and grey literatures in this field, a set of relevant keywords was searched in English and Persian language databases as well as in Google and Google Scholar search engines between 1990 and 2020. These searched keywords and databases are represented in Table 1.

**Table 1:** Databases and search strategy

**PubMed**	**Scopus**	**Cochrane**
ID Search Hits	Search Hits	ID Search Hits
#1 “resource allocation*”[tiab]	(TITLE-ABS-KEY (“resource	#1 “resource
#2 “funding formula*”[tiab]	allocation*”)	allocation*”:ti,ab,kw
#3 “budget formula*”[tiab]	OR TITLE-ABS-KEY (“capitation*”)	#2 “capitation*”:ti,ab,kw
#4 “capitation*”[tiab]	OR TITLE-ABS-KEY (“funding	#3 “funding formula*”:ti,ab,kw
#5 #1 OR #2 OR #3 OR #4	formula*”)	#4 “budget formula*”:ti,ab,kw
#6 “need based*”[tiab]	AND (TITLE-ABS-KEY (“need base*”)	#5 (3-#4)
#7 “risk adjustment*”[tiab]	OR TITLE-ABS-KEY (risk adjustment*))	#6 “need base*”:ti,ab,kw
#8 #6 OR #7	AND (TITLE-ABS-KEY (“health care*”))	#7 “risk adjustment*”::ti,ab,kw
#9 “health care*”[tiab]	AND PUBYEAR > 1995	#8 (2-#7)
#10 “health service*”[tiab]	AND (LIMIT-TO (LANGUAGE,	#9 “health*”:ti,ab,kw
#11 “health system*”[tiab]	“English”))	#10 (#5 AND #8 AND #9)
#12 #9 OR #10 OR #11		Filters applied: From 1990/1/1 to 2020/6/30
#13 #5 AND #8 AND #12		
Filters applied: From 1990/1/1 to 2020/6/30		
**Magiran**	**SID**	**Google Scholar**
“need base resource allocation “	“need base resource allocation”	allintitle:
OR “resource allocation “	OR “resource allocation”	“resource allocation”
OR “resource allocation “	OR “health resources”	OR “capitation”
LANGUAGE: (English OR Persian) AND DOCUMENT TYPES: Article	LANGUAGE: (English OR Persian) AND DOCUMENT TYPES: Article	OR “funding formula” OR “budget formula” AND need
		Filters applied: From 1990 to 2020
		LANGUAGE: (English OR Persian)

### Screening

**Fig. 1:**
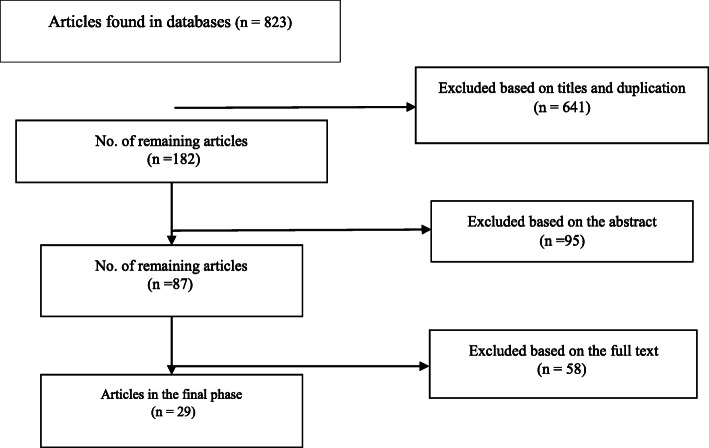
Flow of articles included in the study

A total of 823 articles were extracted from the searched databases. Of them, 641 articles were excluded based on their titles and due to duplication. Afterward, the abstracts of the remaining articles (182) were reviewed, and 95 studies were excluded as well. The full text of the remaining 87 articles was studied, and 58 other articles were then excluded due to their non-compliance with the inclusion criteria of this study. Finally, 29 articles were entered into the final phase of this study (Fig. 1).

### Inclusion and exclusion criteria

Those primary and reviewed published articles (both conference and journal papers) in English or Persian language introducing specific criteria and indicators in the need-based financial resource allocation, were included in this study. Notably, the studies whose abstracts were not in English or Persian or whose full texts were unavailable, were excluded at this stage. A Google search was also performed to find the gray literatures. The items found were then included in the final review if they met the inclusion criteria, so the gray literatures were not ignored and excluded from our systematic review.

### Data extraction and analysis

The information of the selected articles were extracted via a pre-designed form using MAXQDA software (version 10). The data related to each study were extracted independently by two people. In this regard, some minor disagreements between the researchers were resolved by the third person, and the extraction process was finalized if all three people agreed (Table 1).

## Results

Of 823 articles found in the initial search, 29 articles were apprised reviewed and then evaluated in the final analysis. Examining the results of these studies conducted on the need-based allocation of financial resources in the health systems of different countries, has revealed that different indicators and methods are used to allocate financial resources according to the specific conditions of each country. Moreover, it was indicated that the main purposes of all these methods are promoting justice in having access to health services, making the use of these services available, and increasing the efficiency of services. Table 2 indicates the included studies characteristics and Table 3 shows the common indicators used in the distribution of financial resources in the health systems of different countries.

**Table 2:** Included studies characteristics

Authors, year (ref)	Type of Document	Classes of Methodology	Country
Gordon, 2001 [20]	Report	Review	Alberta, Canada, Belgium, England, Finland, France, Germany, Israel, Italy, Netherlands, New South, Wales, Australia, New Zealand, Norway, Scotland, Spain, Stockholm, Sweden, Switzerland, USA, Wales
Shamsi kooshki, 2014 [21]	Article	Review	Alberta, Canada, New South, Wales, Australia, New Zealand, Stockholm, Sweden, USA
Kirigia, 2009 [9]	Thesis	Original research	Alberta, Canada, England, New South Wales, Australia, New Zealand, Scotland, Stockholm, Sweden, Switzerland
Rice, 2001 [10]	Article	Review	Alberta, Canada
Rice, 1999 [22]	Report	Review	Alberta, Canada, Belgium, England, Finland, France, Germany, Israel, Italy, Netherlands, New South, Wales, Australia, New Zealand, Northern Ireland, Norway, Scotland, Spain, Stockholm, Sweden, USA, Wales
Dixon, 2011 [23]	Article	Original research	England
Penno, 2012 [24]	Report	Review & Original research	England, Netherlands, New South Wales, Australia, New Zealand, Ontario Canada, Scotland, Stockholm, Sweden
Buck, 2013 [25]	Report	Review	England
Brick, 2010 [26]	Report	Review & Original research	England
Vega, 2010 [27]	Report	Review & Original research	England, New South, Wales, Australia, New Zealand, Wales Northern Ireland, Scotland, Stockholm, Sweden
Diderichsen, 2004 [11]	Report	Review	England, South Africa, Stockholm, Sweden, Uganda
Nagy, 2015 [28]	Report	Review & Original research	Germany, Netherlands, Stockholm, Sweden
Tidemand, 2008 [29]	Report	Review	Kenya
Briscombe, 2010 [30]	Report	Review	Kenya, South Africa, Tanzania
Manthalu, 2010 [31]	Article	Original research	Malawi
McIntyre, 2012 [8]	Report	Review & Original research	Mozambique, Namibia, Tanzania
McIntyre, 2012 [8]	Report	Review & Original research	Namibia, Zambia, Zimbabwe
McIntyre, 2007 [32]	Report	Original research	Namibia, South Africa, Zambia, Zimbabwe
Ministry of Health and Social Services of Nambia, 2005 [33]	Report	Review & Original research	Namibia
Zere, 2007 [34]	Article	Original research	Namibia
Khan, 2013 [35]	Thesis	Original research	Ontario Canada
Sutton, 2006 [36]	Report	Review & Original research	Scotland
Andersson, 2000 [37]	Article	Original research	Stockholm, Sweden
Anell, 2012 [38]	Report	Review	Stockholm, Sweden
Semali, 2005 [39]	Report	Original research	Tanzania
Pearson, 2002 [40]	Report	Review	Uganda

**Table 3:** Indicators of need-based financial resource allocation in health systems worldwide

Country	Macro indicators of financial resource allocation	Micro indicators of financial resource allocation
Alberta, Canada [9, 10, 20–22]	Age; Sex; Ethnicity; Welfare status	Remoteness; Cross-boundary flows; Funding loss protection; Cost variations
Belgium [20, 22]	Age; Sex; Disability; Unemployment	Urbanization; Mortality
England [9, 11, 20, 22–27]	Age (Sex); Additional Need	Market Forces Factor (staff, land & building costs); Unmet Need; Growth Area Adjustment (rurality, scale economies & case-mix factors)
Finland [20, 22]	Age; Disability	Archipelago; Remoteness; Tax base
France [20, 22]	Age	Phased implementation
Germany [20, 22, 28]	Age; Sex; Invalidity; Morbidity; Sick pay	Income base
Israel [20, 22]	Age	–
Italy [20, 22]	Age; Sex	Mortality; Damping mechanism
Kenya [29, 30]	–	Infrastructure; Under-5 population; Poverty rate; AIDS cases; Females of reproductive age (15 to 49); Area of district (sq. km.)
Malawi [31]	population size; asset indices	–
Mozambique [8]	population size; demographic composition; infant mortality	population density
Namibia [8, 32–34]	population size (weighted by the demographic composition); deprivation index	mortality levels
Netherlands [20, 22, 24, 28]	Age-Sex; Source of Income; Region; Welfare/Disability status; Pharmacy Cost Groups; Diagnosis Cost Groups	Urbanization; Retrospective Adjustments; Income base
New South Wales, Australia [9, 20–22, 24, 27]	Age; Sex; Health Needs Index (HNI); Unavoidable Costs; Unmet need; Indigenous Weight; Homelessness	Teaching and Research; Geographical Adjustment; State-wide Services; Cross-boundary flows; Substitution
New Zealand [9, 20–22, 24, 27]	Age; Sex; Deprivation (Welfare status); Ethnicity	Rurality; Unmet Need; Overseas Visitors; Phased implementation
Northern Ireland [22, 27]	Age; Sex	Mortality; Elderly living alone; Welfare status; Low birth weight; Rural costs adjustment
Norway [20, 22]	Age; Sex; Marital status	Mortality; Elderly living alone; Tax base
Ontario, Canada [24, 35]	Age; Refined Clinical Group; Socioeconomic Status; Rurality	Market Share; Unit Costs; Population Growth
Scotland [9, 20, 22, 24, 27, 36]	Age- Sex; Morbidity and Life Circumstances; Unmet Need	Excess Costs (Remoteness/Rural Cost …)
South Africa [11, 30, 32]	population size; deprivation index	–
Spain [20, 22]	–	Cross-boundary flows; Declining population adjustment
Stockholm, Sweden [9, 11, 20–22, 24, 27, 28, 37, 38]	Age; Cohabitation and marital status; Housing Tenure; Educational Level; Employment Status; Urbanization;	Costly Diagnosis Groups; Phased implementation
Switzerland [9, 20]	Age; Sex; Region	Income base
Tanzania [8, 30, 39]	population size (Age/Sex); under 5 mortality rate; Rurality	poverty level
Uganda [11, 40]	population (age-sex); Human Development Index; per capita donor and NGO spending	security situation
USA [20–22]	Age; Sex; Disability; Welfare status; Previous in-patient diagnosis; county of residence	–
Wales [20, 22, 27]	Age/Sex; Standardized mortality ratio; Additional Need	Extra Costs (Road length, Mean travel Distance...)
Zambia [8, 31]	population size; deprivation index	burden of disease
Zimbabwe [8, 31]	population size; socio-economic status	morbidity and mortality rates; service coverage

As the data in these tables shows, different countries use one or a combination of two general “individual-level data and area-level data” methods, in order to allocate their resources. All of these indicators can be classified into 11 distinct groups as follows: Population size, Demographic indicators, Ethnicity, Socio-economic status, Population mortality rate, Disease complications in the population, Geographical factors, Place of residence, Cross-boundary flow, Costs of providing health services, and Donations by donors. As well, the most commonly used indicators were found to be age, gender, socio-economic status or deprivation, ethnicity, standardized mortality ratio (SMR), the modified health indicators (disease consequences, self-assessed health, and disability), geographical area / place of residence (geographical) (rural versus urban), cross-boundary flows, cost of services, and donations.

Reviewing the existing formulas has also shown that all models performed based on per capita, initially take the advantage of the indexes “population size and the age-sex distribution of the populations of those areas”. In fact, these indexes form the basis for further calculations.

## Discussion

In this study, the commonly used indicators in compiling the formula of allocating financial resources were examined. Need-based formulas, as more equitable allocations of health budgets to geographical areas of a health system, are increasingly used in recent years as an alternative for the historical methods [8, 22, 41, 42]. To develop a need-based formula, it is important to have a practical definition of justice at first [34]. In other words, defining a need-based approach is known as the first essential step in selecting the indicators “necessity and need to compile a formula for allocating need-based resources” [43]. According to Starfield, justice is defined as “No difference in access to health services for equal health needs or greater access for the population defined in terms of social, demographic or geographical statuses with greater health needs” [44]. In addition, the theoretical basis of the need-based allocation formula is that the need for health care in populations with equal size is not necessarily equal, and that the population characteristics are considered as the basis for inferring the population’s relative needs [45–47].

As mentioned earlier in the findings section of this study, the most common need indicators used to measure the relative need for health care services were found as age, gender, socio-economic status or deprivation, ethnicity, standardized mortality ratio (SMR), the modified health indicators (disease consequences, self-assessed health, and disability), geographical area / place of residence (geographical) (rural versus urban), and cross-boundary flows. In this regard, although some indicators such as the cost of services and donations are not considered as need indicators, they are used in the resource allocation formula in some countries.

### Population size

It was indicated that personal characteristics of each individual determine his/her needs for health care services. Owing to the wide variation of the population size in different provinces, the population size in a geographical area is currently considered as the first important indicator of the need for health services in the resource allocation formulas [15, 32, 42, 48].

### Demographic indicators

Population composition in a region or a country (especially age and gender) is a key demographic factor in determining the relative need for health care services in a geographical area, which may be due to a close relationship between age / gender and the need for health care services [15, 22, 42, 49–51].

Therefore, demographic composition can bring more weights in the resource allocation model compared to other factors [52]. In this regard, three main age / gender groups of children, women of reproductive age (childbearing), and the elderly people were found as the most vulnerable population groups to diseases; therefore, they need more health care services [32, 53–55]. Accordingly, the population size in these groups is regarded as an important factor affecting the need for health care services, and consequently, affecting health resources in different regions and areas. For example, the Resource Allocation Working Party (RAWP) claimed that demographic features could affect the need for health care services and weigh the population of each region due to the national use of health services by different age and gender groups [56]. In the British formula, 18 different age groups within health trusts were adjusted through the national use of health services in these trusts [57]. In South Africa [8], the age / sex ratio adjusted based on the national use of health services in each group is usually used as a need factor in the health resource allocation formula. Children aged under 5 years old are selected as a demand criterion for child care services, women aged between 15 and 49 years old are chosen as an indicator of the increased need for health care services (experienced by women who mainly are in childbearing age), and people aged 65 years old and more are recognized as the criterion of the need for the elderly care. According to the data shown in Table 1, it can be seen that the age/ gender factor has been used in all the studied countries (except Israel and France that have only used the age factor).

### Ethnicity

Ethnicity is often used instead of race, citizenship, and country of birth in both matrix and ecological models [11]. In some countries, some ethnic groups do not use health services (e.g. the Maori people in New Zealand and non-Nordic immigrants in Sweden). In New Zealand, it was estimated that how much Maori do not use health care services and thus weighting was adjusted, while ethnicity was not considered in the Swedish model. As a result, it was cleared that this index can be used in a country where different ethnicities are living in different regions.

### Socio-economic status

Socioeconomic status or deprivation is often used as an indirect indicator for the relative need for health care services [22, 34, 41, 45]. Since the relationship between socioeconomic status and the need for health care services is not a simple and straightforward relationship [58], so weighs less than one are assigned to socio-economic factors in a need-based formula. In different countries, various socio-economic indicators are used as indicators of the need for adjusting the models for allocating health care resources; for example, income / assets (Netherlands, South Africa, Malawi), homelessness and education (New South Wales), unemployment (Belgium and Stockholm), welfare status (Alberta, Netherlands, New Zealand, Northern Ireland and USA), marital status (Norway, Stockholm), family structure (Norway), quality of housing (Belgium), house ownership and social class (Stockholm), and cohabitation (Stockholm, Northern Ireland). In South Africa, unemployment, people living under poor housing conditions, lack of access to clear water, poor toilet facilities, lack of access to clean energy sources, illiteracy of the household’s head, and female-headed households have been used as indicators in the resource allocation formula in order to compile socio-economic indicators [41]. Accordingly, Namibia has used a deprivation index using household assets, including electricity, radio, television, refrigerator, and motorcycle; as well as drinking water and toilet type for equitable health resource allocation among its provinces [34].

### Population mortality rate

Owing to some features such as having a familiar concept, reliability, and ease of data collection process, mortality indicator is considered as one of the most common indicators selected to demonstrate the need for health care [48, 59]. Standardized mortality ratio (SMR) and age / sex specific mortality indicators have been also used as indicators for need to know the health resource allocation [16]. For example, raw and standardized mortality ratios have been used as indicators for the need in Per capita schemes in some countries, including Belgium, Italy, Namibia, Northern Ireland, Norway, Scotland Wales, and Zimbabwe. Making the use of mortality indicators may have some disadvantages because the health system provides health services for the living not for the dead, so the resource allocation indicators should be directed to the living as much as possible. Moreover, using some mortality statistics like SMR to allocate resources may not be appropriate in some cases, because a significant portion of health care services is provided for people whose performed treatment did not cause death. Finally, the geographical distribution of health needs may not be in line with the geographical distribution of death caused by diseases in different parts of a country. For example, some people who die in one area may be due to some diseases they contracted in another area some years ago [60]. Since the relationship between mortality and need is not a straightforward relationship, the mortality rate cannot be mechanically considered in the resource allocation formula, as it may lead to some unreasonable and unrealistic allocation patterns [61]. Hence, weighs less than one are applied to this index in the resource allocation formulas [52].

### Disease’s complications in the population

Disease complications are directly resulted from poor health (ill-health) conditions in the population [12]. The prevalence of some chronic diseases such as diabetes, cardiovascular problems, and osteoarthritis as well as the occurrence of some acute complications such as the gastrointestinal and respiratory injuries or infection are the examples of appropriate indicators of disease’s complications, which are considered to assess the need for health care services [16]. In Stockholm, Sweden [37] and the NHS, data on disease’s complications have been used in combination with socioeconomic factors, to allocate health financial resources [59]. Furthermore, self-reported health, which is people’s perception of their health compared to the peers’ health, is considered as an appropriate indicator of disease complications because it is closely related to many other health indicators and independent from the indicator “health services use” [14, 62]. However, making the use of disease’s complications as a need indicator is not popular due to its technical problems. For example, data on disease’s complications may be biased owing to some differences between the records of institutions and regions [14]. In addition, disease’s complication indicators may not cover all the health conditions that people need in order to enjoy health care services [15]. This may underestimate the need for health care resources in areas more requiring these resources. Moreover, there are always some limitations in the frequency, timing or availability of data on disease’s complication for the entire populations and regions; in turn, they impose some restrictions on assessing the need for health through disease’s complications [63].

### Geographical factors

Geographical area is usually used as an indicator to decide on the allocation of resources in most health systems. Some reasons have been given to justify the allocation of resources based on any specific geographical area. For example, making use of the geographical area-based resource allocation approach and differences in the cost of providing health services in different regions, can be counterbalanced by appropriate reimbursements [14]. The United Kingdom, Scotland or Ontario Canada have considered these differences in their formulas. Of note, the resource allocation based on the geographical area has the potential of including both the justice goals and the efficiency goals of health systems [64]. Allocating a larger share of the health budget to geographical areas needing more health care services can increase the efficiency in the use of health services [11]. As well, fair distribution of health credits among geographical areas can improve a previous inappropriate distribution of health facilities and trusts in the regions [65, 66].

### Place of ​​residence

Place of residence (province, cities, towns, urban-rural areas, and slums) can affect both health and chance of improving living conditions [67, 68]. Notably, geographical differences are regarded as an ethical concern in terms of having access to health care services and health outcomes [10]. Therefore, geographical classification is considered as an important tool for promoting health status and properly distributing health resources among different regions [69]. Living in urban or rural areas affects people’s health status in different ways [70]. Urban life provides citizens with many opportunities and better living standards. However, on the other hand, living in urban environments can increase health risks and reshape population health issues from infectious diseases and malnutrition to non-communicable diseases, violence, injuries, and deaths caused by accidents and the effects of environmental disasters [71].

### Cross-boundary flow

Cross-boundary flow is where patients may cross health care boundaries in order to have access to neighboring health services because the required services are not available at their place of residence or due to the reason that there is an unreasonable delay in obtaining care services [31, 72, 73]. Cross-border use of health services is often enjoyed by temporary guests who include people using the facilities provided in the border regions, people seeking their treatments in other cities or abroad, and people referred to other cities or abroad by their health sponsors [72, 74]. Cross-boundary flows are considered as an element in some resource allocation formulas (including Alberta, Canada, New South Wales, and Spain). However, in many cases, there still is a lack of information on cross-boundary flows, especially in developing countries. Accordingly, this consequently places limitations on the inclusion of “cross-border use of health services” in need-based resource allocation formulas [74].

### Costs of providing health services

Costs of providing similar services can greatly vary from each region to another region [74]. For example, costs can be much higher in remote rural areas due to higher transportation costs in these regions and perks given to employees in order to encourage them to travel to these regions. Moreover, owing to a small number of people living in a region or a country with low population density, the cost may be wasted [33]. In addition, due to different input costs, service costs may also vary among buyers [75]. .Altogether, these factors implicitly indicate the need for adjusting a need-based resource allocation formula based on the differences in service provision costs resulted from the effects of geographical factors. However, more appropriate data must be provided to include various costs in the formula. Additionally, making decisions on adjusting different service costs usually is a political issue [11]. Of note, Alberta, the United Kingdom, Ontario, Scotland, the United States, and Wales are the countries that have used this indicator in their formulas.

### Donations by donors

Alternative financial resources provided by donors and NGOs, especially for low-income countries, are recognized as another indicator used in compiling a need-based allocation formula. The challenge posed by this indicator on health policymakers and planners is whether the government should allocate fewer resources in areas with higher donations [40]. In this regard, Uganda is an example of those countries that use this index to allocate their health care budgets according to the following weighting: 60% based on the population size index among different age groups, 20% based on the human development index (per capita income, life expectancy, and school enrollment rates), and 20% based on the donations and NGO expenditures in each region [11].

As mentioned earlier, the geographical area is usually the most common and important decision-making factor for resource allocation in most health systems worldwide; thus, it is known as the basis for the need-based allocation formula because geographical conditions can affect health, thereby affecting the use of health care services [10, 62]. In most of these formulas, the weighted capitation is used in order to estimate the relative need for providing health care services in each geographical area. Concerning the main indicators of population composition (age and gender), especially age (because the gender distribution usually is very similar in different regions) [11], ,socio-economic factors (education or occupation, income, wealth, marital status, and employment status), and geographical factors, this approach ensures more equitable distribution of resources in different geographical areas in terms of the principle “equal access to health care services for all people with the equal need” [8, 10, 11, 42, 62, 76]. Age with greater weight for newborns and population over 75, is the most prominent factor used to pay per capita in high-income countries, while socioeconomic factors as well as those factors associated with disease’s complications are considered as less important criteria, except psychiatric- and society-based care services. However, in low- and middle-income countries, the population aged under 5 years old, poverty indicators and rural population have the highest frequency, which are of great importance in the development of need-based financing formulas [11]. The essential point about the possibility of using these indicators is that they must meet the requirements in order to be used as indicators of the need in developing resource allocation models. Accordingly, the main seven criteria that the “need indicators” must meet are as follows: universally recorded, verifiable, consistent, no incentive for gaming, no vulnerability to manipulation, confidentiality respected, and plausible [10, 14, 15].

In general, as described earlier in the findings section, there are various indicators of the need for health care services. However, there are serious limitations and disagreements related to the selection of indicators owing to the previously emphasized criteria and assumptions, the absence of enough research evidence on the appropriate factors, lack of dependence and legitimacy of the need factors, and lack of proper and relevant information on the potential need indicators [14, 77].

## Conclusion

There are various methods used for need-based resource allocation, which can vary from simple indicators such as population size and composition that are mainly used in developing countries to complex models used in developed countries. Notably, each one of these methods is designed according to the conditions of those countries. Having access to data; the possibility of calculating the index for the given region; values ​​and ethical criteria; and cultural, economic, and social conditions are among the most important factors considered in the allocation of resources. According to the findings of the present study, an appropriate combination of demographic, mortality, and socio-economic indicators with geographical location seems to be effective on developing a need-based allocation formula, thereby improving justice in the distribution of financial resources. Although the allocation of financial resources in health systems seems to be economic in nature, ethical standards of society must be taken into consideration for fairly allocating the resources. Therefore, the most appropriate method of need-based resource allocation in the health system in each country is the one designed or selected as a simple and transparent method, which uses indicators that meet the moral norms of that community and is a good representative of people’s health needs in different geographical areas of that country. Moreover, the information on the characteristics of that model must be available to a great extent.

## Data Availability

The datasets used and/or analysed during the current study are available from the corresponding author on reasonable request**.**

## References

[1] Lotfi F, Rezapour A, Motlagh SN, Hadian M, Faghisolouk F, Ghaderi H. A survey of health sector from the perspective of economics and its relationship with other sectors in Iran's economy. J Health Adm. 2014;17(58).

[2] Sabik LM, Lie RK (2008). Priority setting in health care: lessons from the experiences of eight countries. Int J Equity Health.

[3] Jehu-Appiah C, Baltussen R, Acquah C, Aikins M, d'Almeida SA, Bosu WK (2008). Balancing equity and efficiency in health priorities in Ghana: the use of multicriteria decision analysis. Value Health.

[4] Singer P, Mapa J (1998). Ethics of resource allocation. Hospital Q.

[5] Karimi I, Nasiripour A, Maleki M, Mokhtare H (2006). Assessing financing methods and payment system for health service providers in selected countries: designing a model for Iran. J Health Adm.

[6] Alleyne G, Breman J, Claeson M, Evans D, Jamison D, Jha P (2006). Disease control priorities in developing countries. World Bank/OUP.

[7] Asthana S, Gibson A (2008). Health care equity, health equity and resource allocation: towards a normative approach to achieving the core principles of the NHS. Radic Stat.

[8] Mcintyre D, Anselmi L (2012). Guidance on using needs based formulae and gap analysis in the equitable allocation of health care resources in east and southern Africa.

[9] Kirigia DG (2009). Beyond needs-based health funding: resource allocation and equity at the state and area health service levels in New South Wales–Australia.

[10] Rice N, Smith PC (2001). Capitation and risk adjustment in health care financing: an international progress report. Milbank Q.

[11] Diderichsen F (2004). Resource allocation for health equity: issues and methods.

[12] Kephart G, Asada Y (2009). Need-based resource allocation: different need indicators, different results?. BMC Health Serv Res.

[13] McIntosh T (2010). Population health and health reform: needs-based funding in five provinces. Can Pol Sci Rev.

[14] World Health Organization (2008). Formula funding of health services: learning from experience in some developed countries.

[15] Pbra T (2010). Developing a person based resource allocation formula for allocations to general practices in England.

[16] Keith ER. Assessing need. Module 2. The health Planner's toolkit. Ontario: Ontario Ministry of Health and Long-Term Care, Health Results Team for Information Management; 2006.

[17] Hurley J (2005). Developing needs-based funding formulae using individual-level linked survey and utilization data [electronic resource].

[18] WHO Regional Office for Europe (2012). The European health report: health and health systems Copenhagen.

[19] Arash R (2010). Health care financing and its challenges in Iran.

[20] Gordon D, Lloyd E, Senior M, Rigby J, Shaw M, Shlomo YB (2001). Wales NHS resource allocation review independent report of the research team. Education..

[21] Shamsi Kooshki E, Alipouri Sakha M, Hakimeh M (2014). Health care resources allocation: an ethical perspective. Med Ethics.

[22] Rice N, Smith P. Approaches to capitation and risk adjustment in health care: an international survey. Citeseer; 1999.

[23] Dixon J, Smith P, Gravelle H, Martin S, Bardsley M, Rice N, et al. A person based formula for allocating commissioning funds to general practices in England: development of a statistical model. Bmj. 2011;343(nov22 1):d6608. 10.1136/bmj.d6608. 10.1136/bmj.d6608 PMC3222692 22110252

[24] Penno E, Audas R, Gauld R. The state of the art. An analysis of New Zealand's Population-‐Based Funding Formula for Health Services. Dunedin: Centre for Health Systems University of Otago. 2012:3.

[25] Buck D, Dixon A (2013). Improving the allocation of health resources in England.

[26] Brick A, Nolan A, O’Reilly J, Smith S (2010). Resource allocation, financing and sustainability in health care. Evidence for the expert group on resource allocation and financing in the health sector.

[27] Vega A, O'Shea S, Murrin C, Staines A (2010). Towards the development of a resource allocation model for primary, continuing and community care in the health services-volume 2: Dublin City University.

[28] Nagy B (2015). Improving the allocation of health care resources in Poland.

[29] Tidemand P. Local level service delivery, decentralisation and governance: a comparative study of Uganda, Kenya and Tanzania. Commonw J Local Governance. 2009:144–50. 10.5130/cjlg.v0i0.1093.

[30] Briscombe B, Sharma S, Saunders M. Improving resource allocation in Kenya’s public health sector. Washington; 2010.

[31] Manthalu G, Nkhoma D, Kuyeli S (2010). Simple versus composite indicators of socioeconomic status in resource allocation formulae: the case of the district resource allocation formula in Malawi. BMC Health Serv Res.

[32] McIntyre D, Chitah B, Mabandi M, Masiye F, Mbeeli T, Shamu S (2007). Progress towards equitable health care resource allocation in east and southern Africa.

[33] Ministry of Health and Social Services of Namibia, World Health Organisation, Regional Network for Equity in Health in Southern Africa (EQUINET) (2005). Equity in Health Care in Namibia Towards needs-based allocation formula: Regional Network for Equity in Health in Southern Africa.

[34] Zere E, Mandlhate C, Mbeeli T, Shangula K, Mutirua K, Kapenambili W (2007). Equity in health care in Namibia: developing a needs-based resource allocation formula using principal components analysis. Int J Equity Health.

[35] Khan AI (2013). Resource allocation in the public health sector: current status and future prospects: University of Waterloo.

[36] Sutton M, Elliott B, Ma A, Munro A, Teckle P. Geographic differences in the costs of delivering health services in Scotland: implications for the national resource allocation formula. 2006.

[37] Andersson P-Å, Varde E, Diderichsen F (2000). Modelling of resource allocation to health care authorities in Stockholm County. Health Care Manag Sci.

[38] Anell A, Glenngård AH, Merkur S, Organization WH (2012). Sweden: Health system review.

[39] Semali IA, Minja G (2005). Deprivation and the equitable allocation of health care resources to decentralised districts in Tanzania. Regional Network for Equity in Health in Southern Africa (EQUINET) Equinet Discussion paper.

[40] Pearson M (2002). Allocating public resources for health: developing pro-poor approaches: DFID, Health Systems Resource Centre.

[41] McIntyre D, Muirhead D, Gilson L (2002). Geographic patterns of deprivation in South Africa: informing health equity analyses and public resource allocation strategies. Health Policy Plann.

[42] Health Economics Unit of University of Cape Town, Centre for Health Policy of Wits University (2003). EQUINET methods toolkit: Deprivation and resource allocation: Methods for small area research South Africa: University of Cape Town, University of Witwatersrand.

[43] Østerdal L, Hasmanii A, Hope T (2006). An inquiry into needs-based allocation of health care resources.

[44] Starfield B (2001). Basic concepts in population health and health care. J Epidemiol Community Health.

[45] Birch S, Eyles J. Needs-based planning of health care: a critical appraisal of the literature. Canada: University of Manitoba: Centre for Health Economics and Policy Analysis; 1991.

[46] Birch S, Eyles J, Hurley J, Hutchison B, Chambers S (1993). A needs-based approach to resource allocation in health care. Can Public Policy/Analyse de Politiques.

[47] Ministry of Health and Medical Education, Ministry of Cooperative Labour and Social Welfare (2011). The Family Physician Plan and Refferal System in Iran guideline Tehran.

[48] Oliveira MD, Bevan G (2003). Measuring geographic inequities in the Portuguese health care system: an estimation of hospital care needs. Health Policy.

[49] Martens PJ, Sanderson D, Jebamani LS (2005). Mortality comparisons of first nations to all other Manitobans. Can J Public Health.

[50] Tang KK, Petrie D, Rao D (2007). Measuring health inequalities between genders and age groups with realization of potential life years (RePLY). Bull World Health Organ.

[51] McMunn A, Nazroo J, Breeze E (2009). Inequalities in health at older ages: a longitudinal investigation of the onset of illness and survival effects in England. Age Ageing.

[52] Babaie MH. Inequities in health and health care between provinces of Iran: promoting equitable health care resource allocation. Salford: University of Salford; 2014.

[53] Okojie CE (1994). Gender inequalities of health in the third world. Soc Sci Med.

[54] Mendoza-Sassi R, Béria JU (2001). Health services utilization: a systematic review of related factors. Cadernos de saude publica.

[55] Layte R (2009). Projecting the impact of demographic change on the demand for and delivery of healthcare in Ireland: ESRI.

[56] Smith PC (2008). Resource allocation and purchasing in the health sector: the English experience. Bull World Health Organ.

[57] Iranو TCfP-MitMoHaMEo (2010). Acheivements and Challenges in the Health System of Islamic Republic of Iran1979–2008.

[58] Blackwell DL, Martinez ME, Gentleman JF, Sanmartin C, Berthelot J-M (2009). Socioeconomic status and utilization of health care services in Canada and the United States: findings from a binational health survey. Med Care.

[59] Sutton M (2002). Vertical and horizontal aspects of socio-economic inequity in general practitioner contacts in Scotland. Health Econ.

[60] Yousefi M, Akbari SA, Arab M, Oliaeemanesh A (2010). Methods of resource allocation based on needs in health systems, and exploring the current Iranian resource allocation system. Hakim Res J.

[61] Cengiz M, Ganidagli S, Alatas N, San I, Baysal Z (2008). Partial neuromuscular blockage levels with mivacurium during mastoidectomy allows intraoperative facial nerve monitoring. ORL..

[62] Asante AD, Zwi AB, Ho MT (2006). Equity in resource allocation for health: a comparative study of the Ashanti and northern regions of Ghana. Health Policy.

[63] Vallejo-Torres L, Morris S, Carr-Hill R, Dixon P, Law M, Rice N, et al. Can regional resource shares be based only on prevalence data? An empirical investigation of the proportionality assumption. Soc Sci Med. 2009;69(11):1634–42. 10.1016/j.socscimed.2009.09.020. 10.1016/j.socscimed.2009.09.020 19819058

[64] Mossialos E, Dixon A (2002). Funding health care in Europe: weighing up the options. Funding health care: options for Europe.

[65] Sepehrdoust H (2009). Eliminating health disparities call to action in Iran. Int J Appl Econ Finance.

[66] Kiadaliri AA, Najafi B, Haghparast-Bidgoli H (2011). Geographic distribution of need and access to health care in rural population: an ecological study in Iran. Int J Equity Health.

[67] Marmot M, Friel S, Bell R, Houweling TA, Taylor S (2008). Health CoSDo. Closing the gap in a generation: health equity through action on the social determinants of health. Lancet.

[68] Karunakaran E, Biggs CA (2011). Mechanisms of Bacillus cereus biofilm formation: an investigation of the physicochemical characteristics of cell surfaces and extracellular proteins. Appl Microbiol Biotechnol.

[69] Braveman P (2006). Health disparities and health equity: concepts and measurement. Annu Rev Public Health.

[70] Hartley D (2004). Rural health disparities, population health, and rural culture. Am J Public Health.

[71] Smith LC, Ruel MT, Ndiaye A (2005). Why is child malnutrition lower in urban than in rural areas? Evidence from 36 developing countries. World Dev.

[72] Bertinato L, Busse R, Fahy N, Legido-Quigley H, McKee M, Palm W (2005). Policy brief: cross-border health care in Europe.

[73] Ensor T, Hossain A, Sen PD, Ali L, Begum SA, Moral H. Geographic resource allocation in Bangladesh. Health Policy Res South Asia. 2003;101.

[74] Rich RF, Merrick KR (2006). Cross border health care in the European union: challenges and opportunities. J Contemp Health L & Pol'y.

[75] Scanlon WJ (2006). The future of medicare hospital payment. Health Aff.

[76] Hauck K, Smith PC, Goddard M (2004). The economics of priority setting for health care: a literature review.

[77] Newbold KB, Eyles J, Birch S, Spencer A (1998). Allocating resources in health care: alternative approaches to measuring needs in resource allocation formula in Ontario. Health Place.

